# Peritumoral ductular reaction: a poor postoperative prognostic factor for hepatocellular carcinoma

**DOI:** 10.1186/1471-2407-14-65

**Published:** 2014-02-05

**Authors:** Minhui Xu, Feng Xie, Guangyang Qian, Yingying Jing, Shanshan Zhang, Lu Gao, Tao Zheng, Mengchao Wu, Jiamei Yang, Lixin Wei

**Affiliations:** 1 The Department of General Surgery, The First Affiliated Hospital of Soochow University, Suzhou, China; 2 Tumor Immunology & Gene Therapy Center, Eastern Hepatobiliary Hospital, The Second Military Medical University, Changhai Road, Shanghai 200438, China; 3 Department of Special Treatment and Liver Transplantation, Eastern Hepatobiliary Surgery Hospital, The Second Military Medical University, Shanghai, China

**Keywords:** Ductular reaction (DR), Hepatic progenitor cells (HPCs), Necroinflammation, Fibrosis, Hepatocellular carcinoma (HCC), Prognosis

## Abstract

**Background:**

The role of ductular reaction (DR) in hepatocellular carcinoma (HCC) remains to be elucidated.

**Methods:**

In this study, we tried to uncover possible effect by correlating peritumoral DR in a necroinflammatory microenvironment with postoperative prognosis in HCC. The expression of peritumoral DR/CK19 by immunohistochemistry, necroinflammation and fibrosis were assessed from 106 patients receiving curative resection for HCC. Prognostic values for these and other clinicopathologic factors were evaluated.

**Results:**

Peritumoral DR significantly correlated with necroinflammation (r = 0.563, *p* = 3.4E-10), fibrosis (r = 0.435, *p* = 3.1E-06), AFP level (*p* = 0.010), HBsAg (*p* = 4.9E-4), BCLC stage (*p* = 0.003), TNM stage (*p* = 0.002), multiple nodules (*p* = 0.004), absence of tumor capsule (*p* = 0.027), severe microscopic vascular invasion (p = 0.031) and early recurrence (*p* = 0.010). Increased DR was significantly associated with decreased RFS/OS (*p* = 4.8E-04 and *p* = 2.6E-05, respectively) in univariate analysis and were identified as an independent prognostic factor (HR = 2.380, 95% CI = 1.250-4.534, *p* = 0.008 for RFS; HR = 4.294, 95% CI = 2.255-8.177, *p* = 9.3E-6 for OS) in multivariate analysis.

**Conclusions:**

These results suggested that peritumoral DR in a necroinflammatory microenvironment was a poor prognostic factor for HCC after resection.

## Background

Hepatocellular carcinoma (HCC) is the fifth most common cancer worldwide [1]. Despite the great advancement in diagnosis and treatment modalities, especially surgical and targeted therapies, it’s outcome remains challenging due to frequent recurrence [2]. It is short of effective specific treatment after postoperative metastasis and recurrence in HCC [3]. Therefore, it is of great importance to seek optimal biomarkers that help predict early recurrence or metastasis.

HCC with progenitor cell features, possibly reflecting a progenitor cell origin, has a very bad prognosis [4]. Hepatic progenitor cells (HPCs) exhibit large nuclear-cytoplasmic ratio and oval-shaped nucleus, known as oval cells in rodents [5]. It is believed that HPCs are the descendants of stem cells. HPCs are scarce in the healthy liver, but upon stimulation, cells resident in the Canals of Hering proliferate across the hepatic lobule infiltrating the liver parenchyma [6]. HPCs can be observed by immunohistochemistry and electron microscope. The neoplastic cells are offspring of HPCs and each can differentiate a little differently, according to the local microenvironment in each part of the tumor if it explains the enormous phenotypic heterogeneity of a neoplasm [4]. In immunohistochemistry the phenotypes of HPCs express as OV6, CK7, CK19, CD133 and EPCAM [7-9]. HPCs are verified to be able to differentiate into both hepatocytes and cholangiocytes when the latter fails to respond after severe injury [10].

HPCs form ductular reaction (DR), emanating from the portal zone and expanding into the parenchyma when they are activated to proliferate/differentiate, hepatocytic differentiation of these cells leads to the formation of intermediate hepatocytes [5]. Three types of DR are classically recognized, type 1, proliferation of pre-existing ducts and ductules; type 2, ductular metaplasia of hepatocytes and type 3, activation/proliferation of HPCs [11]. DR can be marked by immunostained with CK7 and CK19 [12,13]. DR plays an important role in hepatocellular or cholangiocellular proliferation after virus related inflammation and damage. DR may represent a protective mechanism that allows intrahepatic cycling of bile acids to occur in chronic ductopenic biliary diseases [13]. Its immunostaining may help to identify small foci of invasion and to distinguish noninvasive, high-grade dysplastic nodules from both minimally invasive and overtly invasive HCC [12].

Activated and proliferative mechanism of HPCs is not clear, and inflammatory cytokine is considered as a key role in animal experiment [14]. Severity and location of inflammatory infiltration associated with activity and location of HPCs in chronic virus hepatitis [15]. Inflammation has emerged as the seventh hallmark of cancer [16]. Such prolonged self-replication in an inflammatory microenvironment could result in the accumulation of genetic lesions that cause cancer formation [17]. There is substantial evidence that the proinflammatory response at the tumor stroma could be rerouted into a tumor-promoting direction by stimulating angiogenesis and tissue remodeling [18].

However, the role of peritumoral DR in a necroinflammatory microenvironment remains to be elucidated in HCC. At the present study, we investigated DR and necroinflammatory microenvironment of patients with HCC. We also tried to uncover possible effect by correlating DR in a necroinflammatory microenvironment with postoperative prognosis in HCC.

## Methods

### Patients and specimens

106 patients received curative resection of HCC in Eastern Hepatobiliary Hospital, the Second Military Medical University between 2001 and 2003. The total number of the patients with resected tumors during the same time period was 2180. Patients did not have signs of distant metastasis nor had they received anticancer therapy before surgery. The pathology of each patient was confirmed. Liver function was assigned by Child-Pugh scoring system. The role of the child pugh score was that 2 patients were B and 104 patients were A in this study. The tumor stage was determined according to 2002 AJCC/UICC tumor-node-metastasis (TNM) classification system and the Barcelona-Clinic Liver Cancer (BCLC) staging classification. Follow-up was as described in our previous report [1]. Data was censored at the last follow-up for patients without recurrence or death. Recurrence-free survival time (RFS) and overall survival time (OS) was defined as the interval between the time of surgery to that of recurrence or death, respectively. All human sample collection procedures were approved by China Ethical Review Committee and informed consent was obtained from all participants.

### H&E and immunohistochemistry

The paraffin-embedded tissues stained with H&E were scored in a blinded manner according to Ishak scoring system by a single pathologist. The degree of necroinflammatory activity and the stage of fibrosis were scored 0–18 or 0–6 respectively in the non-tumor specimen according to Ishak *et al.*[19]. Median values were used as a cut-off in subsequent analyses. Immunohistochemistry was carried out according to appropriate protocols [20]. The primary antibody used was mouse monoclonal anti-CK19 (1:100, clone RCK108, Dako), Blank controls were treated identically except that the primary antibodies were omitted.

### Evaluation of DR and morphometric determinations

CK19-immunoreactive DR was analyzed at the epithelial-stromal boundaries at the outer edge of tumor. Peritumoral DR was semiquantified as follows: 0 = none, 1 = <10%, 2 = 10% to 25%, 3 = 26% to 50%, and 4 = >50% [12]. Median value was used as a cut-off in subsequent analyses. HPCs and intermediate hepatobiliary cells were considered as CK19-positive cells, previous finding showing that [5].

### Tissue samples

The study was approved by the Committee on Ethics, the Eastern Hepatobiliary Surgery Hospital of the Second Military Medical University, informed consent which has been conducted according to the principles expressed in the Declaration of Helsinki was obtained from each patient. All participants provided their written informed consent to participate in this study. HCC tissues were obtained from patients who underwent surgical operations for the tumors at Eastern Hepatobiliary Surgery Hospital.

### Statistical analyses

Correlations between immunostaining parameters and clinicopathologic features were analyzed by χ2 tests, the Fisher’s exact test, and Spearman’s rho coefficient test as appropriate. Univariate and multivariate analysis was carried out with the Kaplan-Meier method and the Cox proportional hazards regression model and was compared with the log-rank test. For each analysis, only *p* <0.05 (two-sided) was considered statistically significant. All statistical analyses were made by use of SPSS 12.0 (SPSS Inc., Chicago, IL, USA).

## Results

### H&E and immunohistochemical characteristics

**Figure 1:**
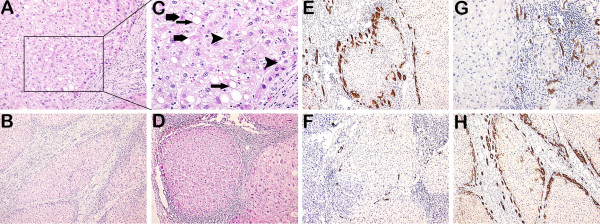
**Immunostaining of DR, histopathology of necroinflammation and fibrosis/cirrhosis. (A, C)** Ballooning degeneration (arrowheads), steatosis (thin arrows) and focal necrosis (thick arrows) were shown in the peritumoral parenchyma. **(B)** Cirrhosis and interface hepatitis were shown. **(D)** Fibrosis/cirrhosis and portal inflammation were shown. **(E)** Increased DR was around a nodule in the peritumoral tissue. **(F)** Decreased DR was around a nodule in the peritumoral tissue. **(G)** DR and necroinflammation were shown. Ballooning degeneration, steatosis and focal necrosis were nearby DR. Inflammatory cells were around DR in the portal tracts. **(H)** DR and cirrhosis were shown. DR was at the periphery of a cirrhotic nodule. (**A** and **G** 200×; **C** 400×; **B**, **D**, **E**, **F** and **H** 100×).

**Figure 2:**
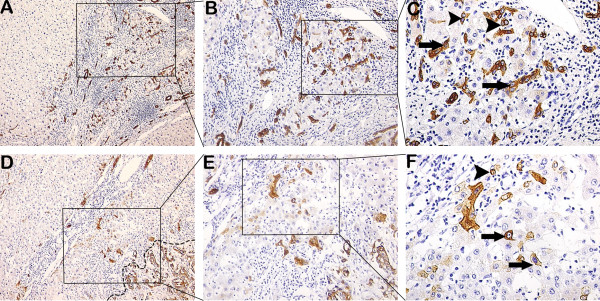
**Immunostaining of DR, histopathology of HPCs and intermediate hepatocytes in fibrotic tissue or peritumoral and tumoral tissues. (A, B, C)** DR emanated from the portal zone and expanded into the parenchyma of proliferative nodules, HPCs (arrowheads) and intermediate hepatocytes (arrows) can be seen in the nodules. **(D, E, F)** DR and CK-19 positive tumor (in dashed line) that had no capsule were shown. HPC (arrowhead) was close to DR, intermediate hepatocytes (arrows) were between DR and neoplastic nodule. (**A** and **D** 100×; **B** and **E** 200×; **C** and **F** 400×).

**Figure 3:**
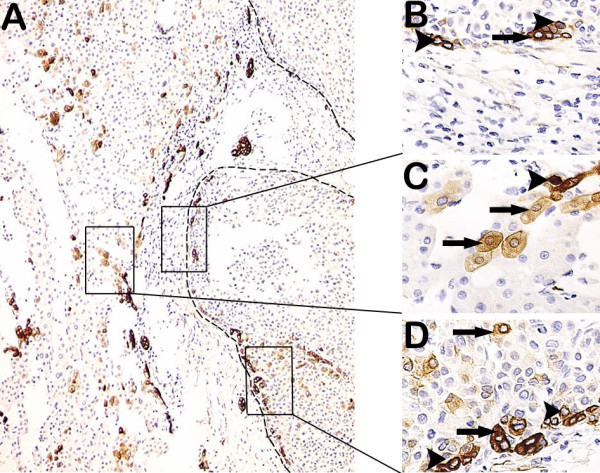
**Correlations of DR with HPCs and intermediate hepatocytes in the peritumoral and tumoral tissues. (A)** DR was at the periphery of the portal tracts. CK-19 positive tumor (in dashed line) had a partial capsule. A lot of intermediate hepatocytes were diffused in the peritumoral parenchyma. **(B)** At the tumoral borderline, 2 or 5 HPCs (arrowheads) gathered tightly, one intermediate hepatocyte (arrow) was among them. **(C)** In the peritumoral tissue, several intermediate hepatocytes (arrows) were nearby DR that was at the periphery of the portal tracts, and HPC (arrowhead) was in DR. **(D)** At the tumoral borderline, HPCs (arrowheads) gathered together, one intermediate hepatocyte (arrow) was closed to them, while intermediate hepatocytes (arrows) were diffused in the tumoral parenchyma. (**A** 100×; **B**, **C** and **D** 400×).

Representative images of necroinflammation and fibrosis/cirrhosis are shown (Figure 1). DR is shown in Figures 1, 2 and 3. As shown in Table 1, DR significantly correlated with necroinflammatory grade (r = 0.563, *p* = 3.4E-10) and fibrotic stage (r = 0.435, *p* = 3.1E-06). HPCs, intermediate hepatocytes and correlations with DR are shown in Figures 2 and 3.

**Table 1:** Necroinflammatory grade, fibrotic stage and correlations with DR

**Variable**	**Median**	**25 and 75 percentiles**	**Range**	**Correlations with DR**	
				**r**	***p***
DR	2	2-3	1-4	N.A.	
Necroinflammatory grade	9	7-12	4-15	0.563	3.4E-10
Fibrotic stage	4	3-5	1-6	0.435	3.1E-06

Correlations of DR with necroinflammatory grade and fibrotic stage were tested using Spearman’s rho coefficients tests.

### Correlations between DR and clinicopathologic features

As shown in Table 2, increased DR correlated with advanced BCLC stage (*p* = 0.003), TNM stage (*p* = 0.002), with elevated serum ALT (*p* = 0.017), ALP (*p* = 0.007), AFP (*p* = 0.010) and HBsAg (+) (*p* = 4.9E-4). Increased DR was also tended to correlate with multiple nodules (*p* = 0.004), absence of tumor capsule (*p* = 0.027), severe microscopic vascular invasion (*p* = 0.031) and early recurrence (*p* = 0.010).

**Table 2:** Correlations of DR with clinicopathologic features

			**DR (<2 vs ≥2)**		
**Variable**			**Low (%)**	**High (%)**	***p***
Age (year) (median; 25 and 75 percentiles)	50; 40-59	≤50	13 (50.0)	43 (53.8)	0.379
		>50	13 (50.0)	37 (46.2)	
Gender		Male	22 (84.6)	66 (82.5)	0.534
		Female	4 (15.4)	14 (17.5)	
ALT (U/L) (median; 25 and 75 percentiles)	44.6; 29.9-72	≤40	16 (61.5)	28 (35.0)	0.017
		>40	10 (38.5)	52 (65.0)	
γ-GT (U/L) (median; 25 and 75 percentiles)	74.6; 46.4-106.8	≤55	13 (50.0)	24 (30.0)	0.063
		>55	13 (50.0)	56 (70.0)	
ALP (U/L) (median; 25 and 75 percentiles)	145; 116.5-190	≤121	13 (50.0)	18 (22.5)	0.007
		>121	13 (50.0)	62 (77.5)	
AFP (ng/ml) (median; 25 and 75 percentiles)	325; 40.9-1000	≤20	11 (42.3)	14 (17.5)	0.010
		>20	15 (57.7)	66 (82.5)	
HBsAg state		Negative	10 (38.5)	6 (7.5)	4.9E-04
		Positive	16 (61.5)	74 (92.5)	
Tumor size (cm) (median; 25 and 75 percentiles)	7; 4.6-11.8	≤5	7 (26.9)	28 (35.0)	0.447
		>5	19 (73.1)	52 (65.0)	
Tumor number		Single	25 (96.2)	54 (67.5)	0.004
		Multiple	1 (3.8)	26 (32.5)	
Tumor capsule		Yes	10 (38.5)	14 (17.5)	0.027
		None	16 (61.5)	66 (82.5)	
Vascular invasion		No	18 (69.2)	52 (65.0)	0.692
		Yes	8 (30.8)	28 (35.0)	
Microscopic vascular invasion (mean ± SD)*	4.3 ± 3.5	≤5	7 (87.5)	10 (35.7)	0.031
		>5	1 (12.5)	18 (64.3)	
BCLC stage (A/B/C)	39/12/55	A	16 (61.5)	23 (28.8)	0.003
		B/C	10 (38.5)	57 (71.2)	
TNM stage (I/II/III)	35/23/48	I	15 (57.7)	20 (25.0)	0.002
		II/III	11 (42.3)	60 (75.0)	
Recurrence**		Early	8 (47.1)	52 (65.0)	0.010
		Late	9 (52.9)	12 (35.0)	

Chi-square tests for all the analyses. *Vascular invasion positive cases (36) were enrolled, the degree of microscopic peritumoral vascular invasion was observed. **A total of 81/106 (76.4%) patients suffered from tumor recurrence, of whom 60/81 (74.1%) patients recurred within two years and 21/81 (26.0%) recurred more than two years after surgery.

### Prognostic factors

The follow-up was completed on December 25, 2009, with median follow-up time of 93 months (75 to 107 months). The 1-, 3-, 5-, 7-year RFS and OS rates were 54.7%, 24.5%, 19.8%, 8.5% and 71.7%, 36.8%, 32.1%, 20.8% respectively.

**Figure 4:**
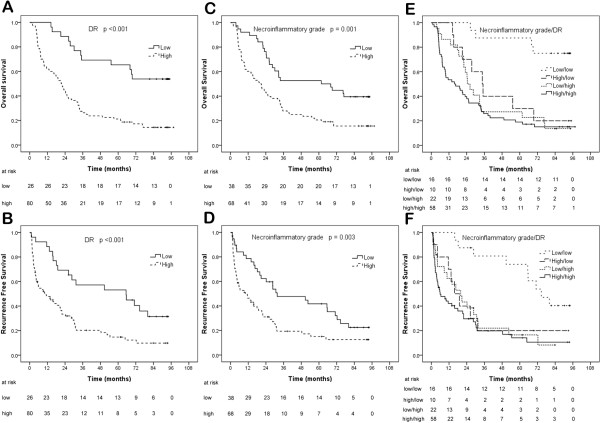
**Kaplan–Meier analysis of DR and necroinflammatory grade from original cohort.** Decreased DR **(A and B)**, decreased necroinflammatory grade **(C and D)** and decreased DR with decreased necroinflammatory grade **(E and F)** were significantly associated with prolonged RFS (low/low vs high/low, low/high and high/high, *p* = 0.013, = 0.001 and <0.001; high/low vs low/high, *p* = 0.858; high/low vs high/high, *p* = 0.347; low/high vs high/high, *p* = 0.356) and OS (low/low vs high/low, low/high and high/high, *p* = 0.002, <0.001 and <0.001; high/low vs low/high, *p* = 0.472; high/low vs high/high, *p* = 0.191; low/high vs high/high, *p* = 0.373). The median was used as cut-off of DR and necroinflammatory grade, respectively.

In the univariate analysis, decreased DR (*p* = 2.6E-05 for OS and *p* = 4.8E-04 for RFS, Figure 4A and B), decreased necroflammation (*p* = 0.001 for OS and *p* = 0.003 for RFS, Figure 4C and D) and decreased DR with lower necroflammation (*p* = 6.1E-05 for OS and *p* = 0.001 for RFS, Figure 4E and F) were associated with prolonged RFS and OS. Other clinicopathologic factors significant for RFS/OS are shown in Table 3.

**Table 3:** Univariate and multivariate analyses of prognostic factors

**Variable**	**Overall survival**			**Recurrence-free survival**		
	**Univariate**	**Multivariate**		**Univariate**	**Multivariate**	
	***p***	**HR (95% CI)**	***p***	***p***	**HR (95% CI)**	***p***
Age, year (≤50 vs >50)	0.719		N.A.	0.881		N.A.
Gender (female vs male)	0.013		N.A.	0.003		N.A
ALT, U/L (≤40 vs >40)	0.264		N.A.	0.800		N.A.
γ-GT, U/L (≤55 vs >55)	0.030		N.A.	0.581		N.A.
ALP, U/L (≤121 vs >121)	0.007		N.A	0.077		N.A
Serum albumin, g/L (<35 vs ≥35)	0.632		N.A	0.712		N.A
AFP, ng/ml (≤20 vs >20)	0.095		N.A.	0.169		N.A.
HBsAg (negative vs positive)	0.207		N.A.	0.449		N.A.
Tumor size, cm (≤5 vs >5)	6.0E-05	2.950 (1.694-5.137)	1.3E-04	0.003	1.804 (1.057-3.080)	0.031
Tumor number (single vs multiple)	0.006		N.A.	3.3E-05		N.A.
Tumor capsule (yes vs none)	0.016		N.A.	0.015		N.A.
Microscopical intrahepatic metastasis (no vs yes)	0.165		N.A.	0.021		N.A.
Microscopic vascular invasion (no vs yes)	0.354		N.A.	0.017		N.A.
Portal vein invasion (no vs yes)	0.004		N.A.	0.348		N.A.
BCLC stage (A vs B/C)	3.3E-08	2.738 (1.602-4.679)	2.3E-04	2.2E-07		N.A.
TNM stage (I vs II /III)	1.8E-07		N.A.	3.4E-08	3.597 (2.046-6.324)	8.8E-06
Necroinflammatory grade (<9 vs ≥9)	0.001		N.A.	0.003	1.837 (1.087-3.105)	0.023
Fibrotic stage (≤4 vs >4)	0.014		N.A.	0.103		N.A.
DR (<2 vs ≥2)	2.6E-05	4.294 (2.255-8.177)	9.3E-06	4.8E-04	2.380 (1.250-4.534)	0.008
Liver ischemic time, min (≤25 vs >25)	0.760		N.A.	0.683		N.A.
Blood loss, ml (<800 vs ≥800)	0.007		N.A.	0.063		N.A.
Blood transfusion (yes vs no)	0.089		N.A.	0.071		N.A.

The median was used as cut-off of necroinflammatory grade, fibrotic stage and DR respectively. Variables with *p* <0.05 in univariate analysis were adopted for multivariate analysis. Tumor size and DR were included, other covariates including tumor number, tumor capsule and portal vein invasion were then excluded.

Factors demonstrated to be significant in univariate analysis were then used into the Cox proportional hazards regression model for multivariate analysis. Both DR and tumor size were independent prognostic factors for RFS and OS. DR was associated with elevated risks of recurrence (HR = 2.380, 95% CI = 1.250-4.534, *p* = 0.008) and death (HR = 4.294, 95% CI = 2.255-8.177, *p* = 9.3E-06). Tumor size was associated with elevated risks of recurrence (HR = 1.804, 95% CI = 1.057-3.080, *p* = 0.031) and death (HR = 2.950, 95% CI = 1.694-5.137, *p* = 1.3E-04). Besides, TNM and necroinflammatory grade were demonstrated as independent predictors for RFS; while BCLC stage was independent prognostic factors for OS.

There are two types of recurrence for HCC, one is early recurrence (true metastasis, ≤2 years after surgery) and the other is late recurrence (*de novo* cancer, >2 years after surgery) [21]. A total of 81/106 (76.4%) patients suffered from tumor recurrence, of whom 60/81 (74.1%) patients recurred within two years and 21/81 (26.0%) recurred more than two years after surgery. As shown in Table 2, patients with high DR were more likely to suffer from early recurrence compared with the low subgroup (8/52 vs 9/12, *p* = 0.010).

## Discussion

To the best of our knowledge, this is the first study to identify peritumoral DR as an independent prognostic factor for HCC after resection. Patients with decreased peritumoral DR had a significantly prolonged OS and RFS compared with the increased subgroup. Therefore, patients with high peritumoral DR require closer follow-up after surgery, and peritumoral DR could also serve as a new biomarker predicting HCC recurrence.

DR occurs in cholestatic diseases, in inflammatory diseases and in conditions with massive loss of parenchyma [22-24]. Hence, three types of DR are classically recognized [11]. We precluded the DR which originated from bile ducts obstructive diseases or cholestatic parenchymal damage in 106 patients with HCC, so the DR we observed could represent an activation of HPCs. Intermediate hepatobiliary cells of several differentiation states are continuously being produced in a dynamic nature of DR [25]. These data are supported by our results (Figures 2 and 3). DR is thought to arise due to a complex interaction between hepatocytes, hepatic progenitor cells, hepatic stellate cells and extracellular matrix as well as inflammatory cells and endothelial cells [13]. The total necroinflammatory score was significantly associated with the expansion of DR (Table 1). The result is in agreement with recent findings [26]. There was a highly significant correlation between the area of DR and fibrotic stage [27]. It may be important in the development of fibrosis [28]. These data are supported by our result showing that DR was significantly correlated with fibrotic stage (Table 1). Insulin resistance and hepatic inflammation might cause liver fibrosis by the expansion of the DR and the occurrence of epithelial-mesenchymal transition (EMT) [27-29]. Our data showed that HPCs were in or closed to DR (Figures 2 and 3). Other authors proposed that DR is an important source of progenitor cells that can repopulate both the bile ductular and hepatocytic lineages in diseased liver [5,30].

HPCs expansion forms DR by provided “field-effects” of a continuing necroinflammatory microenvironment. They were evidenced by their location in close proximity to the DR, and the strong correlation between these two variables [27]. The combination of interferon-γ and tumor necrosis factor alpha, cytokines overexpressed in HCV (Hepatitis C virus) may inhibit primary hepatocyte replication and stimulate HPCs expansion [31-33]. Cellular signalling between HPCs and the surrounding nonparenchymal population is an important determinant of HPCs behaviour [34]. Our subsequent result showed that DR was higher in HBsAg (+) subgroup than in HBsAg (−), which meant that HPCs in DR expanded actively in HBsAg (+) (Table 2). ECM remodelling, such as fibrosis resolution and laminin deposition is likely to be important prerequisite to HPCs activation and expansion [35]. The collected evidence indicates that HPCs, in a suitable environment, could be induced to a direction of portal fibroblastic differentiation through EMT [36]. Chronic carbon tetrachloride (CCl_4_) administration to mice induces significant hepatic fibrosis and can induce a florid HPCs response in parallel with advanced fibrosis [37]. It was suggested that the HPC response may drive liver fibrogenesis rather than being a secondary event [38].

Inflammation can supply bioactive molecules to the tumor microenvironment, including limiting cancer cell death [39,40]. Necrotic cell death releases proinflammatory signals into the surrounding tissue microenvironment. As a consequence, necrotic cells can recruit inflammatory cells of the immune system [39,41]. Additionally, necrotic cells can release bioactive regulatory factors, such as IL-1a, which can directly stimulate neighboring viable cells to proliferate [39]. Incipient neoplasias and potentially invasive and metastatic tumors may gain an advantage by tolerating some degree of necrotic cell death, doing so in order to recruit tumor-promoting inflammatory cells that bring growth-stimulating factors to the surviving cells within these growths [42].

DR expressing CK perhaps contains ones deriving from malignant degeneration of HPCs. The CK-positive HCC cells play the leading role in directing aggressive behavior of the tumor cell population [43]. The presence of CK19 expression by >5% of cells in HCC is associated with a poor prognosis [8]. The study of CK expression in the liver provides a useful insight into the mechanisms underlying progenitor cell activity and tissue regeneration following liver damage. HPCs express CK7 and CK19 [44]. They potentially derive from malignant degeneration of HPCs. HCC expressing CK19 had a higher incidence of AFP expression [8]. Our data showed that increased CK19-immunoreactive DR correlated with elevated serum AFP (Table 2). The higher recurrence rate of CK19 (+) HCC after transplantation suggests a worse prognosis for HCC expressing CK19 as compared to CK19 (−) HCC [8].

There are two types of recurrence for HCC: one is early recurrence, the other is late recurrence [21]. Discriminating between these two types is necessary in order to determine the appropriate intervention after surgery. True metastasis would benefit from adjuvant chemotherapy, while *de novo* cancer would be prevented by better control of viral infection and/or cirrhosis [45]. Our data showed that patients with early recurrence had significantly higher DR compared with patients with late recurrence. Patients with recurrence had especially higher DR and high necroinflammation compared with patients without recurrence, indicating the importance of alleviating necroinflammation, protecting hepatic function and strengthening general immunity. DR correlates with the degree of inflammation and fibrosis in the course of many chronic human liver diseases. Inflammation can supply bioactive molecules to the tumor microenvironment that facilitates inductive signals that lead to activation of EMT [39]. Some results indicated the critical association between the metastasis and EMT [46]. Mesenchymal stem cells in inflammation microenvironment accelerate HCC Metastasis by Inducing EMT [47]. DR may undergo the process of EMT in patients with the highest grade of necroinflammation [26]. Maybe the higher peritumoral DR was the prognostic indicator for early recurrence after hepatectomy due to intrahepatic metastasis through EMT.

The lack of liver donation, highly cost of operation and post-operative treatment, caused that few liver transplantation was carried out in our hospital. OS rates after hepatectomy for HCC was low. 5-yr survival after hepatectomy for HCC was 32.1% in this series. Because there were 71 (67%) patients with HCC whose tumor size was more than 5 cm, and the tumor size was independent prognostic factors for RFS and OS in our study. On the other hand, 67 (63.2%) patients had B/C of BCLC stage, 71 (67%) patients had II/III of TNM stage. Our study showed that increased peritumoral DR often correlated with multiple nodules and the absence of a tumor capsule, which are two features of a highly invasive HCC phenotype. Our study also showed that increased peritumoral DR correlated with severe microscopic vascular invasion. The cases with peritumoral multiple portal vein invasions tend to show early recurrence after hepatic resection. That’s the reason why it is common in early recurrence. Therefore, a very close follow-up protocol is required for patients with increased DR because it usually reveals highly aggressive tumor behavior.

## Conclusion

In conclusion, we demonstrated for the first time that peritumoral DR in a necroinflammatory microenvironment is a poor prognostic factor for HCC following Resection. A continuing necroinflammatory microenvironment provides “field-effects” for stimulating HPCs expansion to form DR and partly to be diverted to malignant direction. DR expressing CK19 perhaps contains ones deriving from malignant degeneration of HPCs. Maybe the pretumoral DR is the prognostic indicator for early recurrence after hepatectomy due to intrahepatic metastasis through EMT. This provides a rationale for anti-inflammatory, anti-EMT and HPCs targeted therapies in clinical practice. Further experiments are needed to reveal the mechanisms of DR, EMT and inflammatory cells.

## Abbreviations

AFP: Alpha fetoprotein; BCLC stage: Barcelona-Clinic Liver Cancer stage; CCl4: Chronic carbon tetrachloride; DR: Ductular reaction; EMT: Epithelial-mesenchymal transition; HCC: Hepatocellular carcinoma; HCV: Hepatitis C virus; HPCs: Hepatic progenitor cells; OS: Overall survival time; RFS: Recurrence-free survival time.

## Competing interests

The authors have declared that no competing interests exist.

## Authors’ contributions

JiY and LW designed the research; MX, FX, YJ, SZ, LG, and TZ performed the research; MX, GQ and MW analyzed the data; and MX and FX wrote the paper. All authors read and approved the final manuscript.

## Pre-publication history

The pre-publication history for this paper can be accessed here:

http://www.biomedcentral.com/1471-2407/14/65/prepub
